# *In Vivo* Persistence of Human Rhinoviruses in Immunosuppressed Patients

**DOI:** 10.1371/journal.pone.0170774

**Published:** 2017-02-02

**Authors:** Ilka Engelmann, Anny Dewilde, Mouna Lazrek, Mathilde Batteux, Aminati Hamissi, Ibrahim Yakoub-Agha, Didier Hober

**Affiliations:** 1 Laboratoire de Virologie EA 3610, Faculté de Médecine, Université Lille et CHU Lille, Lille, France; 2 Maladies du Sang, CHU Lille et Faculté de Médecine, Université Lille, Lille, France; 3 INSERM U995, LIRIC, Lille, France; University of Hong Kong, HONG KONG

## Abstract

Several species of the genus *Enterovirus* cause persistent infections in humans. Human rhinovirus (HRV) infections are generally self-limiting but occasionally persistent infections have been described. This study aimed to identify persistent HRV infections and investigate the clinical and virologic characteristics of patients with persistent infections. From January 2012 to March 2015, 3714 respiratory specimens from 2608 patients were tested for respiratory viruses by using a multiplex reverse transcription–polymerase chain reaction. A retrospective study was performed. Patients with at least two specimens positive for HRV/enterovirus taken 45 days or longer apart were identified and the HRV/enteroviruses were typed. Patients with persistent infection were compared to patients with reinfection and patients with cleared infection. Phylogenetic analysis of the viral protein(VP)4/VP2 region was performed. 18 patients with persistent HRV/enterovirus infection were identified. Minimum median duration of persistence was 92 days (range 50–455 days). All but one patients with persistence were immunosuppressed. Immunosuppression and hematologic disorders were more frequent in patients with persistence (n = 18) than in patients with reinfection (n = 33) and with cleared infection (n = 25) (p = 0.003 and p = 0.001, respectively). In conclusion, this retrospective study identified HRV persistence in vivo which occurred mainly in immunosuppressed patients.

## Introduction

Human rhinoviruses (HRV) are classified into three species (A, B and C) within the genus *Enterovirus* of the family *Picornaviridae* [1,2]. HRVs mainly cause upper respiratory tract infections. However, they can also cause lower respiratory infection and are associated with exacerbations of chronic pulmonary diseases [3], such as asthma [4], chronic obstructive pulmonary disease [5] and cystic fibrosis [2,6].

Viral persistence has been described for several *Enterovirus* species, and it was linked to clinical entities, e.g. persistent infection with poliovirus is associated with post-polio syndrome and persistent infection with Coxsackievirus is associated with chronic myocarditis and dilatative cardiomyopathy [7,8,9,10,11,12,13,14]. A possible link of persistent enterovirus infection and type 1 diabetes is also strongly suspected because several studies detected enteroviral RNA and/or viral proteins in peripheral blood and/or pancreatic tissue of patients with type 1 diabetes [15,16,17,18].

HRV infection usually presents as an acute infection with viral shedding of up to 42 days as shown by experimental HRV infection [19]. A recent study followed healthy infants in the first year of life and found that HRV RNA rarely persisted beyond 30 days after HRV infection [20]. Persistent HRV infection has been occasionally described in immunosuppressed patients, namely transplant recipients [21,22,23] and patients with hypogammaglobulinemia [24,25] but to date it is unclear how widespread the phenomenon of HRV persistence is. In fact, in a previous study we found no evidence for HRV persistence in asthmatic children [4].

The objective of the present study was to identify persistent HRV/enterovirus infections and to investigate virologic and clinical characteristics of these. To address this objective, patients with at least two specimens positive for HRV/enterovirus taken 45 days or longer apart were identified retrospectively and the HRV/enteroviruses were typed in order to determine whether it was an infection with the same HRV/enterovirus type (persistent infection) or a reinfection with a different type.

## Methods

### Patients and specimens

From January 2^nd^ 2012 to March 22^nd^ 2015, 3714 respiratory specimens from 2608 patients were sent to the laboratory of virology of Lille University Hospital Center for testing in a multiplex reverse transcription-polymerase chain reaction (RT PCR) for respiratory viruses. The number of patients with 1, 2 or more specimens tested is shown in S1 Table. The diagnostics for respiratory viruses was prescribed by the physician in charge of the patient. In our hospital centre, no systematic virological surveillance is performed for respiratory viruses, even in severely immunocompromised patients, such as stem cell transplant recipients. Therefore all patients had at least one respiratory symptom or fever.

Demographic data (age, sex) and data on underlying diseases and immunosuppression were retrospectively obtained from hospital records.

### Ethics statement

The study was carried out in accordance with the Declaration of Helsinki and was approved by the institutional review board (Comité de Protection des Personnes Nord Ouest IV) with waiver of informed consent. Therefore informed consent was not obtained. It was a retrospective, noninterventional study with no additional procedures. Specimens were initially sent by the physicians for diagnostic testing for respiratory viruses.

### Definitions

#### Persistent HRV/enterovirus infection

**Fig 1:**
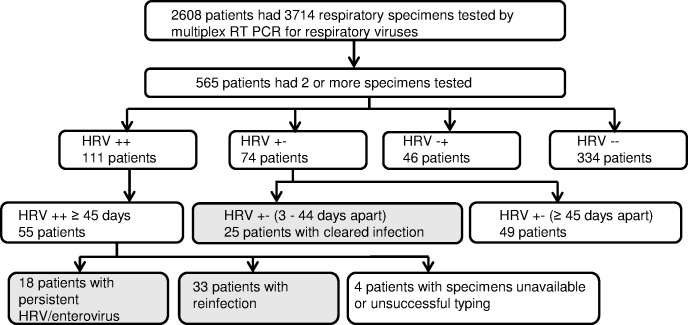
Flow diagram for patients and specimens included in the study.

Patients were included in the “persistent infection” group if they had detection of the same HRV/enterovirus type (defined as >/ = 90% nucleic acid sequence identity [1]) in two or more respiratory specimens that were taken at an interval of 45 days or more (HRV++ ≥ 45 days) (Fig 1). The time interval of 45 days was chosen because normally, the duration of viral shedding after experimental HRV infection is at most 42 days [19]. In case the same HRV/enterovirus type was detected in the first and the last specimen of a patient, specimens obtained in between those were not systematically submitted to typing. However, if the HRV/enterovirus type differed between the first and the last specimen of a patient, specimens obtained in between those were submitted to typing. A patient who was included in the “persistent infection” group was not included in the “reinfection” group even if he/she had an episode of reinfection with another HRV type.

#### Reinfection

Patients were included in the “reinfection” group if they had detection of different HRV/enterovirus types in two or more respiratory specimens that were taken at an interval of 45 days or more (HRV++ ≥ 45 days) (Fig 1).

Cleared infection: Patients were included in the “cleared infection” group if they had a respiratory specimen with detection of HRV/enterovirus followed by a specimen without HRV/enterovirus detection taken between 3 and 44 days after the positive specimen (HRV +- (3–44 days apart)) (Fig 1).

**Infectious episode:** infection with the same HRV/enterovirus type.

**URT:** Upper respiratory tract specimens (URT) are nasopharyngeal swabs or aspirates.

**LRT:** Lower respiratory tract specimens (LRT) are bronchoalveolar lavage fluids, bronchial or tracheal aspirates.

HRV++: detection of HRV/enterovirus in two or more respiratory specimens
HRV++ ≥ 45 days: detection of HRV/enterovirus in two or more respiratory specimens that were taken at an interval of 45 days or more
HRV +-: specimen with detection of HRV/enterovirus followed by a specimen without HRV/enterovirus detection
HRV +- (3–44 days apart): specimen with detection of HRV/enterovirus followed by a specimen without HRV/enterovirus detection taken between 3 and 44 days after the positive specimen
HRV +- (≥ 45 days apart): specimen with detection of HRV/enterovirus followed by a specimen without HRV/enterovirus detection taken 45 days or more after the positive specimen
HRV-+: specimen without HRV/enterovirus detection followed by a specimen with HRV/enterovirus detection
HRV--: all specimens were negative for HRV/enterovirus

### Detection of respiratory viruses

Respiratory specimens were stored at -80°C before RNA extraction. RNA was extracted using the Magtration System 12GC using the MagDEA Viral DNA/RNA 200 (GC) kit (Precision System Science Co., Ltd.; Japan) according to the manufacturer’s instructions. RNA extracts were submitted to a commercially available multiplex RT-PCR that screens for a panel of respiratory viral pathogens including influenza virus A and B, respiratory syncytial virus A and B, human adenovirus, human metapneumovirus, coronavirus 229E/NL63 and OC43, parainfluenza virus 1–3, HRV A/B (Seeplex RV12 ACE Detection, Seegene, Seoul, Korea) from January to September 2012 (235 specimens, 6.3%) and to a commercially available multiplex RT-PCR that screens for a panel of respiratory viral pathogens including influenza virus A and B, respiratory syncytial virus A and B, human adenovirus (HAdV), human metapneumovirus, coronaviruses 229E,NL63 and OC43, parainfluenza virus 1–4, HRV A/B/C, enterovirus, and bocavirus 1–4 (Anyplex II RV16 Detection, Seegene, Seoul, Korea) from October 2012 to March 2015 (3479 specimens, 93.7%). According to the manufacturer, the detection limit for sensitivity of the RV16 assay is 50 copies/reaction, corresponding to 3125 copies/mL of respiratory specimen. The detection limit of the RV12 assay is not indicated by the manufacturer.

An aliquot of respiratory specimens was stored at -80°C and used for typing.

### Molecular typing of HRV/enterovirus

HRV/enterovirus positive specimens were typed by amplification and sequencing of the viral protein(VP)4/VP2 region using the primers described by Wisdom et al. [26] Briefly, reverse transcription and amplification was performed using the SuperScript III One-Step RT-PCR System with Platinum Taq DNA Polymerase (Invitrogen, Thermofisher Scientific). The mix contained 7.5 μL H_2_0, 25 μL 2× Reaction Mix, 37.5 pmol of primers VP4 OS and VP4 OAS, 2 μL of RT/Platinum Taq Mix and 8 μL of extraced nucleic acids. The RT PCR was performed with the following thermal profile: 30 minutes (min) at 45°C, 2 min at 94°C followed by 4 cycles of 30 seconds (s) at 94°C, 30s at 46°C and 1 min 30s at 68°C, followed by 36 cycles of 30s at 94°C, 30s at 53°C and 1 min 30s at 68°C with a final extension for 7 min at 68°C. Nested PCR was performed using Amplitaq DNA Polymerase with Buffer I (Applied Biosystems, Thermofisher Scientific). 5 μL of the RT PCR product were added to a mix containing 5 μL of buffer I, 2 μL dNTP, 1 μL Amplitaq DNA Polymerase, 0.5 mM MgCl_2_, 28.5 μL H_2_O, 37.5 pmol of primers VP4 IS and VP4 IAS. Reactions were performed with the following thermal profile: 5 min at 94°C followed by 4 cycles of 30s at 94°C, 30s at 46°C and 1 min 30s at 72°C, followed by 34 cycles of 30s at 94°C, 30s at 53°C and 1 min 30s at 72°C with a final extension for 10 min at 72°C. The PCR product was sequenced directly after verification that amplification had yielded a PCR product of the correct size on an agarose gel.

### Sequence and phylogenetic analysis

Sequences were assembled and aligned to HRV/enterovirus genomes by using SeqScape Software v2.7 (Applied Biosystems). Consensus sequences were submitted to the Basic Local Alignment Search Tool (BLAST; http://blast.ncbi.nlm.nih.gov) using the blastn algorithm in order to identify the nearest HRV/enterovirus type.

Additionally, sequences were aligned and phylogenetic trees were constructed using Mega-Software (MEGA 6 [27]) using both neighbour joining and maximum likelihood methods including reference sequences for each HRV/enterovirus type to confirm the nearest HRV/enterovirus type.

**Fig 2:**
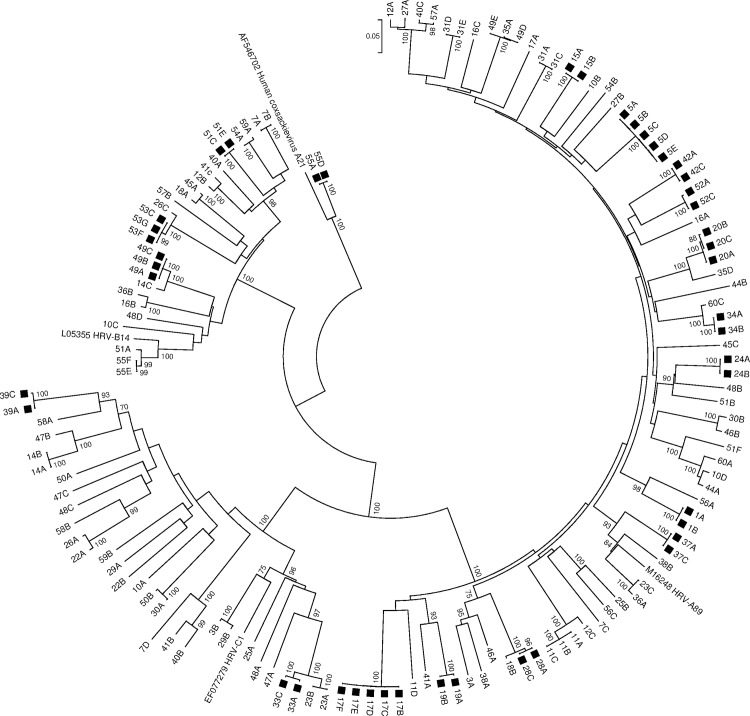
Phylogenetic analysis of the nucleotide sequences of the HRV/enterovirus VP4/VP2 region.

A phylogenetic tree limited to the specimens from this study and one reference sequence each for HRV-A, -B and -C as well as Coxsackievirus A21 was constructed (Fig 2) by using Mega-Software (MEGA 6 [27]) and the neighbour joining method with maximum composite likelihood model and pairwise deletions. Bootstrapping was performed with 1000 replicates.

A neighbour joining tree of partial VP4/VP2 sequences was constructed by using MEGA 6 [27]. The numbers indicate the patients and the letters the specimens, with A being the first chronologically. Coxsackievirus A21 was used as out-group. The percentage of bootstraps (out of 1000) that supports the corresponding clade is shown at the nodes if higher than 70%. HRV-A, -B and -C and Coxsackievirus A21 reference sequences were included. The scale bar indicates nucleotide substitutions per site. Persistent infections are indicated with a black square.

GenBank accession numbers of the sequences obtained in this study are indicated in S1 Text.

In patient 53, the first two specimens were unavailable for typing, therefore only specimens 53C to G are reported.

### Statistical analysis

Statistical analysis was performed by using IBM SPSS Statistics 22 (IBM, United States) and an online statistical tool (http://in-silico.net/tools/statistics/fisher_exact_test). A p-value of <0.05 was considered significant. Continuous non-parametric variables were compared using Kruskal-Wallis test and post-hoc tests as appropriate. Categorical variables were compared by using Pearson’s chi-square or Fisher’s exact test as appropriate. If the difference between the three groups was significant, pairwise comparisons were performed and Bonferroni correction was applied to adjust for multiple comparisons. Logistic regression with stepwise backward variable selection was performed comparing the persistence group with a combined group including both patients with cleared infection and reinfection. The following variables with p-value < 0.05 in univariable analysis were included in logistic regression analysis: hematologic disorder, immunosuppression and age. A significance level of 0.1 was chosen for inclusion of variables into a logistic regression model and for remaining included in the model.

## Results

565 patients had two or more specimens tested (Fig 1). Of these, 111 patients had 2 or more specimens that were tested positive for HRV/enterovirus (HRV++), 74 patients had a specimen that tested positive for HRV/enterovirus followed by a specimen that tested negative for HRV/enterovirus (HRV+-), 46 patients had a specimen that tested negative for HRV/enterovirus followed by a specimen that tested positive for HRV/enterovirus (HRV-+) and in 334 patients all specimens were tested negative for HRV/enterovirus (HRV--) (Fig 1). The details of the distribution of these patient groups with regard to the total number of specimens tested per patient is shown in S2 Table. Of the 111 patients that had 2 or more specimens that tested positive for HRV/enterovirus (HRV++), 55 patients had repeated detection of HRV/enterovirus in respiratory specimens taken at least 45 days apart. Specimens of 52 patients were available for typing. Typing was successful in 137 of 139 (98.6%) specimens from 52 patients. Of these, 77 were HRV-A (56.2%), 28 were HRV-B (20.4%), 30 were HRV-C (21.9%) and 2 were Coxsackievirus A21 (1.5%). One patient, in whom the second specimen was untypeable, was excluded from further analysis. Phylogenetic analysis of the VP4/VP2 region of the 136 HRV/enterovirus positive specimens of the other 51 patients is shown in Fig 2.

18 out of 51 patients (35%) had detection of the same HRV/enterovirus type in at least two respiratory specimens taken at least 45 days apart and thus fullfilled the criteria of persistent HRV/enterovirus infection (Fig 1). Underlying conditions in patients with persistent infection were acute myeloid leukaemia (n = 5), acute lymphoblastic leukaemia (n = 3), chronic lymphocytic leukaemia (n = 2), multiple myeloma (n = 1), hemophagocytic syndrome (n = 1), bone marrow aplasia (n = 1), myelofibrosis (n = 1), myelodysplastic syndrome (n = 1), Hodgkin's disease (n = 1), polyarteritis nodosa (n = 1) and cystic fibrosis (n = 1). 10/18 (55.6%) patients with persistent infection had undergone allogeneic stem cell transplantation. Among the 18 patients with persistent infection, the minimum median duration of HRV/enterovirus shedding (i.e. the time interval between the first and the last specimen with the same HRV type) was 92 days (range 50 to 455 days).

33 patients had a reinfection with a different HRV/enterovirus type (Fig 1). 25 patients had a HRV/enterovirus positive specimen followed by a HRV/enterovirus negative specimens at an interval of 3 to 44 days. These patients were included in the cleared infection group (Fig 1). Clinical characteristics of the three groups are shown in Table 1. Age was significantly different between the three groups (p = 0.002, Table 1). Immunosuppression and hematologic disorders as underlying conditions were associated with persistent infection (p = 0.003 and p = 0.001, respectively, Table 1). The variables age and hematologic disorder were associated with persistent infection in logistic regression analaysis.

**Table 1:** Characteristics of patients with and without persistent HRV/enterovirus infection.

Characteristic	Persistent infection,	Reinfection,	Cleared infection,	p-value^a^
	n = 18 [number (%)]	n = 33 [number (%)]	n = 25 [number (%)]	
**Sex, male**	9 (50.0)	19 (57.6)	9 (36.0)	0.26
**Age, years** [median (range)]	40.5 (1–78)	7 (0–63)	3 (0–64)	0.002^b^
**Number of specimens** [median (range)]	4.5 (2–11)	6 (2–10)	2 (2–6)	<0.001^c^
**Underlying condition**				
Hematologic disorder^d^	16 (88.9)	16 (48.5)	8 (32.0)	0.001^e^
Heart disease	0 (0)	3 (9.1)	1 (4.0)	0.55*
Lung disease^f^	1 (5.6)	8 (24.2)	8 (32.0)	0.10*
Cancer	0 (0)	2 (6.1)	0 (0)	0.50*
Other^g^	1 (5.6)	4 (12.1)	7 (28.0)	0.15*
Premature birth	0 (0)	1 (3.0)	4 (16.0)	0.09*
**Immunosuppression**	17 (94.4)	19 (57.6)	11 (44.0)	0.003^h^
**Hospital admission**	11 (61.1)	24 (72.7)	19 (76.0)	0.55
**Other respiratory viruses detected**^**i**^	3 (16.7)	5 (15.2)	5 (20.0)	0.93*

^a^ Chi Square test unless otherwise indicated.

* Fisher’s exact test.

^b^ Kruskal-Wallis test; post-hoc test showed that age is different between the cleared infection versus persistent infection groups (p = 0.005) and reinfection versus persistent infection groups (p = 0.004) but not between the cleared infection versus reinfection groups (p = 1.00).

^c^ Kruskal-Wallis test; post-hoc test showed that the number of specimens is different between the cleared infection versus persistent infection groups (p = 0.02) and cleared infection versus reinfection groups (p<0.001) but not between the reinfection versus persistent infection groups (p = 1.00).

^d^ Hematologic disorders were acute myeloid leukemia (n = 10), acute lymphoblastic leukemia (n = 9), chronic lymphocytic leukemia (n = 2), chronic myeloid leukemia (n = 1), chronic myelo-monocytic leukemia (n = 2), multiple myeloma (n = 3), myelodysplastic syndrome (n = 2), Hodgkin's disease (n = 2), follicular lymphoma (n = 1), hemophagocytic syndrome (n = 1), bone marrow aplasia (n = 2), myelofibrosis (n = 2), auto-immune hemolytic anaemia (n = 1), osteopetrosis (n = 1), Blackfan Diamond syndrome (n = 1).

^e^ Pairwise comparisons showed that there was a significant difference between the cleared infection versus persistent infection groups (p<0.001) and reinfection versus persistent infection groups (p = 0.004) but not between the cleared infection versus reinfection groups (p = 0.207).

^f^ cystic fibrosis (n = 6), bronchopulmonary dysplasia (n = 5), pulmonary agenesis (n = 1), pulmonary valve agenesis (n = 1), follicular bronchiolitis(n = 1), asthma (n = 1), Wegener’s granulomatosis (n = 1), myopathia with history of tracheotomia and long-term mechanical ventilation (n = 1).

^g^ Diaphragmatic hernia (n = 2), polyarteritis nodosa (n = 1), Gorlin syndrome (n = 1), Pompe disease (n = 1), Niemann-Pick C disease (n = 1), myopathia with history of tracheotomia and long-term mechanical ventilation (n = 1), nephrotic syndrome (n = 1), glycogenosis (n = 1), newborn with benzodiazepine severage (n = 1), resection of small intestine and bronchopulmonary dysplasia (n = 1), neurofibromatosis type 1 (n = 1).

^h^ Pairwise comparisons showed that there was a significant difference between the cleared infection versus persistent infection groups (p = 0.001) and reinfection versus persistent infection groups (p = 0.006) but not between the cleared infection versus reinfection groups (p = 0.306).

^i^ Detection of other respiratory viruses at the first or first persistent episode.

The detection of other respiratory viruses was relatively frequent in all three groups (Table 1).

The nearest HRV/enterovirus type, the duration of virus shedding, the respiratory site of the HRV/enterovirus detection and other respiratory viruses that were detected are shown in S3 Table for the 18 patients with persistent infection. HRV-A was detected in most patients with persistent infection (66.7%). When first episodes of infection or persistent infection were compared between reinfection and persistent infection groups, respectively, there was no statistically significant association between HRV/enterovirus species and persistence (p = 0.15) (Table 2).

**Table 2:** Infectious episodes^a^ of persistent HRV/enterovirus infections and reinfections.

	Persistent infection,	Reinfection,
	n = 18 [number (%)]	n = 33 [number (%)]
HRV-A	12 (66.7)	15 (45.5)
HRV-B	3 (16.7)	6 (18.2)
HRV-C	2 (11.1)	12 (36.4)
Coxsackievirus A21	1 (5.6)	0 (0)

^a^The first episode was taken into account for the reinfection group and the first persistent episode was taken into account for the persistent infection group.

## Discussion

In the present study, we have systematically searched for HRV/enterovirus persistence and identified 18 patients with persistent HRV/enterovirus infection. To our knowledge, this is the largest case series of persistent HRV/enterovirus infections so far published. Most patients in this study were hospitalized (54/76, 71.1%) and immunosuppression was frequent (47/76, 61.8%). All but one patients with persistent infection were immunosuppressed, and immunosuppression was associated with HRV/enterovirus persistence (p = 0.003, Table 1). The only patient with persistent infection who was not immunosuppressed suffered from cystic fibrosis. To our knowledge there is only one other report of HRV persistence in a cystic fibrosis patient, namely a HRV-A infection in a 43 year-old-patient [28]. In contrast, our patient had an infection with HRV-B69. Hematologic disorders were the underlying condition in most patients with HRV/enterovirus persistence and statistically associated with persistence (p = 0.001, Table 1). Interestingly, a recent study found HRV persistence in 8 paediatric hematopoietic stem cell transplant recipients [29]. In our study, 10 of the 18 patients (55.6%) with persistent infection were stem cell transplant recipients, 1 child and 9 adults. This population of highly immunosuppressed patients seems to be predisposed for HRV/enterovirus persistence. The minimum median duration of HRV/enterovirus persistence in our patients was 92 days, ranging from 50 to 455 days. To our knowledge, a longer duration of persistence has so far only been described twice [23,28].

Other respiratory viruses were frequently detected in respiratory specimens of patients with persistent HRV/enterovirus infection, patients with reinfection and patients with cleared infection (Table 1, S3 Table). This confirms the findings by Loeffelholz et al [30], although, in our study, the most commonly concomitantly detected viruses were not coronaviruses but human adenoviruses (S3 Table).

Interestingly, most persistent infections were caused by HRV-A. However, no statistically significant association between the species of HRV and persistence was found. This may be due to the relatively small number of patients with persistent infection.

This retrospective study has the following limitations:

Specimens were sent for the diagnostics of respiratory viruses upon prescription of the physician in charge of the patient. Although systematic surveillance for respiratory viruses is not performed at our centre, there is possibly a bias in so far as the physician probably prescribes more readily diagnostics for respiratory viruses in patients that he/she judges at increased risk. That may have led to an overrepresentation of immunosuppressed patients in our study population. However, the fact that immunosuppressed patients probably had more specimens sent for diagnostics for respiratory viruses does not mean that these were necessarily persistent infections. This is supported by the fact that specimen number was not significantly different between the reinfection and persistence groups (Table 1). However, there was a significant difference in specimen number per patient between the cleared infection group versus the other two groups (Table 1).

The second respiratory specimen was obtained at various time intervals following the first HRV/enterovirus positive specimen. Persistence was assumed in patients who harboured the same HRV/enterovirus type in consecutive specimens even if these were taken at a long time interval because reinfection with the same type seems extremely unlikely due to host immunity and due to the high number of circulating types of HRV. In patients in whom we did not find evidence for persistence (i.e. those who had a different HRV type in the second specimen), we do not have data on the duration of the infection with the different HRV types. This is a point that has to be addressed in future studies as well as the clinical relevance of HRV persistence. This is important, because, like persistence of other enteroviruses, HRV persistence might be associated with chronic diseases, for example diabetes type 1 or dilatative cardiomyopathy. The fact that the second respiratory specimen was obtained at various time intervals following the first HRV/enterovirus positive specimen also impacted the “cleared infection” group. Only 25 patients had a negative follow-up specimen that was taken in the time interval of 3 to 44 days after the first specimen and thus permitted to classify them as “cleared infection”. 49 patients had a negative follow-up specimen that was taken 45 days or longer after the first specimen. Thus a persistent infection (according to our definition) could not be excluded and therefore these patients were not included in the “cleared infection” group.

Two different assays for the detection of respiratory viruses were used during the study period. The Seeplex RV12 ACE Detection assay does not include the detection of HRV-C, therefore HRV-C infections were probably not diagnosed in the 235 specimens were tested with this assay. These specimens represent 6.3% of all specimens of this study.

In conclusion, this retrospective study identified 18 patients with HRV/enterovirus persistence in vivo. Persistence occurred mainly in immunosuppressed patients.

## Supporting Information

S1 Table Patient and specimen numbers. (DOC)

S2 Table HRV status and number of specimens tested per patient. (DOC)

S3 Table Virologic characteristics of persistent HRV/enterovirus infections. (DOC)

S1 Text GenBank accession numbers of the sequences obtained in this study. (DOC)

## Abbreviations

HRV: human rhinovirus
VP: viral protein
RSV: respiratory syncytial virus
CoxA21: Coxsackievirus A21
