# Correction: *In Vivo* Persistence of Human Rhinoviruses in Immunosuppressed Patients

**DOI:** 10.1371/journal.pone.0181296

**Published:** 2017-07-13

**Authors:** The PLOS ONE Staff

The legends for Figs 1 and 2 incorrectly appear within the body of the article. The legend for Fig 1 incorrectly appears above the "Detection of respiratory viruses" heading of the Methods section. The legend for Fig 2 incorrectly appears above the “Statistical analysis” heading of the Methods section. There are errors in the caption for Fig 1. Please see the complete, correct captions for Figs 1 and 2 here. The publisher apologizes for the error.

**Fig 1:**
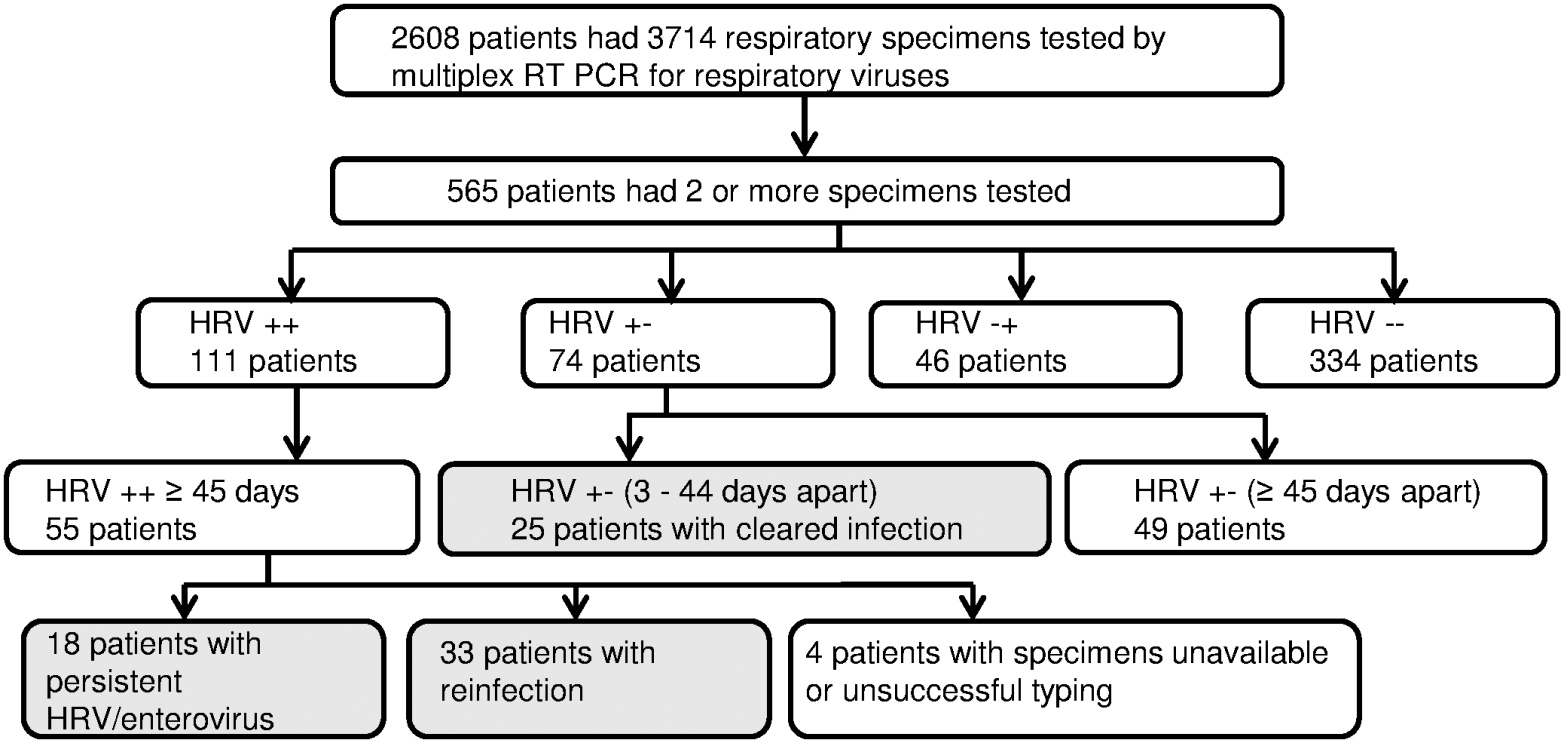
Flow diagram for patients and specimens included in the study. HRV++: detection of HRV/enterovirus in two or more respiratory specimensHRV++ ≥ 45 days: detection of HRV/enterovirus in two or more respiratory specimens that were taken at an interval of 45 days or moreHRV +-: specimen with detection of HRV/enterovirus followed by a specimen without HRV/enterovirus detectionHRV +- (3–44 days apart): specimen with detection of HRV/enterovirus followed by a specimen without HRV/enterovirus detection taken between 3 and 44 days after the positive specimenHRV +- (≥ 45 days apart): specimen with detection of HRV/enterovirus followed by a specimen without HRV/enterovirus detection taken 45 days or more after the positive specimenHRV-+: specimen without HRV/enterovirus detection followed by a specimen with HRV/enterovirus detectionHRV--: all specimens were negative for HRV/enterovirus Grey shading indicates the groups that are shown in table 1.

**Fig 2:**
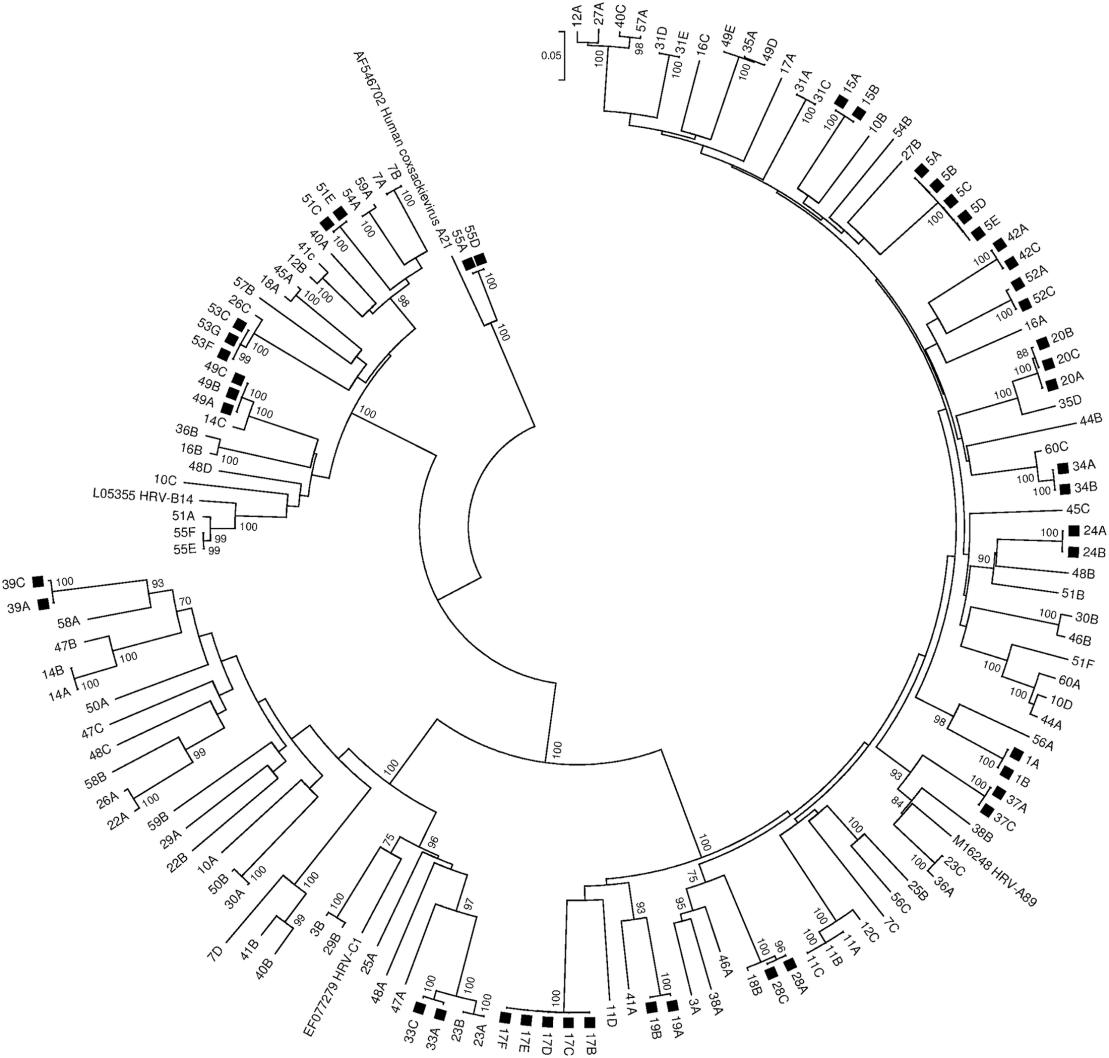
Phylogenetic analysis of the nucleotide sequences of the HRV/enterovirus VP4/VP2 region. A neighbour joining tree of partial VP4/VP2 sequences was constructed by using MEGA 6 [27]. The numbers indicate the patients and the letters the specimens, with A being the first chronologically. Coxsackievirus A21 was used as out-group. The percentage of bootstraps (out of 1000) that supports the corresponding clade is shown at the nodes if higher than 70%. HRV-A, -B and -C and Coxsackievirus A21 reference sequences were included. The scale bar indicates nucleotide substitutions per site. Persistent infections are indicated with a black square. GenBank accession numbers of the sequences obtained in this study are indicated in S1 Text. In patient 53, the first two specimens were unavailable for typing, therefore only specimens 53C to G are reported.
