# Supplementary material for: In Vivo Persistence of Human Rhinoviruses in Immunosuppressed Patients
Source: PLoS One. 2017 Feb 2;12(2):e0170774. doi: 10.1371/journal.pone.0170774 (PMC5289482; doi:10.1371/journal.pone.0170774)
Supplement: S1 Table — (DOC) [file pone.0170774.s001.doc]

**S1 Table. Patient and specimen numbers**

| Number of specimens | Number of patients |
| --- | --- |
| 1 | 2043 |
| 2 | 321 |
| 3 | 119 |
| 4 | 52 |
| 5 | 28 |
| 6 | 16 |
| 7 | 13 |
| 8 | 10 |
| 9 | 3 |
| 10 | 2 |
| 11 | 1 |
| Total 3714 | 2608 |
