# Supplementary material for: In Vivo Persistence of Human Rhinoviruses in Immunosuppressed Patients
Source: PLoS One. 2017 Feb 2;12(2):e0170774. doi: 10.1371/journal.pone.0170774 (PMC5289482; doi:10.1371/journal.pone.0170774)
Supplement: S2 Table — (DOC) [file pone.0170774.s002.doc]

**S2 Table. HRV status and number of specimens tested per patient**

| Number of specimens per patient | Number of patients | HRV++ | | | | | HRV+- | | HRV-+ | HRV-- |
| --- | --- | --- | --- | --- | --- | --- | --- | --- | --- | --- |
|  |  |  | HRV++ ≥ 45 days | | | |  | HRV+-  3-44 days |  |  |
|  |  |  | All | Persistence | | Reinfection |  | (Cleared infection) |  |  |
| 2 | 321 | 40 | 8^a^ | 3 | 4 | | 39 | 13 | 31 | 211 |
| 3 | 119 | 17 | 8^a^ | 4 | 3 | | 13 | 3 | 11 | 78 |
| 4 | 52 | 13 | 7 | 2 | 5 | | 9 | 2 | 3 | 27 |
| >4 | 73 | 41 | 32^b^ | 9 | 21 | | 13 | 7 | 1 | 18 |
| All | 565 | 111 | 55 | 18 | 33 | | 74 | 25 | 46 | 334 |

^a^ 1 patient was excluded because specimens were unavailable for typing

^b^ 2 patients were excluded because specimens were unavailable for typing

HRV++: at least two specimens were positive for HRV/enterovirus

HRV+-: a specimen was positive for HRV/enterovirus was followed by a specimen negative for HRV/enterovirus

HRV-+ : a specimen was negative for HRV/enterovirus was followed by a specimen positive for HRV/enterovirus

HRV--: all specimens were negative for HRV/enterovirus
