# Supplementary material for: In Vivo Persistence of Human Rhinoviruses in Immunosuppressed Patients
Source: PLoS One. 2017 Feb 2;12(2):e0170774. doi: 10.1371/journal.pone.0170774 (PMC5289482; doi:10.1371/journal.pone.0170774)
Supplement: S3 Table — (DOC) [file pone.0170774.s003.doc]

**S3 Table. Virologic characteristics of persistent HRV/enterovirus infections**

| Patient/Specimen number^a^ | Cumulative days | Nearest HRV/enterovirus type  (% identity to nearest type)^b^ | Duration of infection with same type  (% identity to prior sequence)^c^ | Site of HRV/enterovirus detection | Other respiratory viruses |
| --- | --- | --- | --- | --- | --- |
| 1A |  | A23 (98) |  | LRT | Human adenovirus |
| 1B | 56 | A23 (98) | 56 (100) | URT | Coronavirus 229E and OC43, Bocavirus |
|  |  |  |  |  |  |
| 5A |  | A73 (92) |  | URT | None |
| 5B | 18 | A73 (92) | 18 (100) | URT | None |
| 5C | 67 | A73 (92) | 67 (99.7) | URT | None |
| 5D | 102 | A73 (92) | 102 (100) | URT | None |
| 5E | 120 | A73 (92) | 120 (99.7) | URT | None |
|  |  |  |  |  |  |
| 15A |  | A60 (99) |  | LRT | None |
| 15B | 455 | A60 (99) | 455 (100) | LRT | None |
|  |  |  |  |  |  |
| 17A |  | A94 (90) |  | URT | None |
| 17B | 69 | A51 (99) |  | URT | None |
| 17C | 158 | A51 (99) | 89 (100) | URT | None |
| 17D | 173 | A51 (99) | 104 (100) | URT | None |
| 17E | 214 | A51 (99) | 145 (100) | LRT | Human adenovirus |
| 17F | 254 | A51 (99) | 185 (100) | LRT | None |
|  |  |  |  |  |  |
| 19A |  | A20 (99) |  | LRT | None |
| 19B | 97 | A20 (99) | 97 (99.7) | LRT | None |
|  |  |  |  |  |  |
| 20A |  | A43 (98) |  | URT | None |
| 20B | 90 | A43 (98) | 90 (99.2) | URT | None |
| 20C | 257 | A43 (98) | 257 (99.7) | URT | None |
|  |  |  |  |  |  |
| 24A |  | A32 (90) |  | URT | RSV |
| 24B | 223 | A32 (90) | 223 (100) | LRT | None |
|  |  |  |  |  |  |
| 28A |  | A28 (97) |  | LRT | None |
| 28B | 30 | not done |  | URT | None |
| 28C | 86 | A28 (97) | 86 (100) | LRT | Human adenovirus |
|  |  |  |  |  |  |
| 33A |  | C43 (98) |  | URT | Human adenovirus |
| 33B | 46 | not done |  | URT | Human adenovirus |
| 33C | 62 | C43 (98) | 62 (99.5) | LRT | Human adenovirus |
|  |  |  |  |  |  |
| 34A |  | A56 (98) |  | URT | None |
| 34B | 132 | A56 (98) | 132 (100) | LRT | None |
|  |  |  |  |  |  |
| 37A |  | A89 (87) |  | LRT | None |
| 37B | 275 | not done |  | URT | None |
| 37C | 276 | A89 (87) | 276 (100) | LRT | None |
|  |  |  |  |  |  |
| 39A |  | C18 (93) |  | URT | None |
| 39B | 17 | not done |  | URT | None |
| 39C | 108 | C18 (93) | 108 (99.5) | URT | None |
|  |  |  |  |  |  |
| 42A |  | A24 (98) |  | URT | None |
| 42B | 5 | not done |  | LRT | None |
| 42C | 70 | A24 (98) | 70 (100) | LRT | None |
|  |  |  |  |  |  |
| 49A |  | B86 (89) |  | URT | None |
| 49B | 22 | B86 (89) |  | URT | None |
| 49C | 50 | B86 (89) | 50 (100) | URT | Human adenovirus |
| 49D | 119 | A63 (97) |  | URT | None |
| 49E | 141 | A63 (97) |  | URT | None |
|  |  |  |  |  |  |
| 51A |  | B14 (99) |  | URT | None |
| 51B | 152 | A09 (98) |  | URT | None |
| 51C | 358 | B69 (94) |  | URT | None |
| 51D | 400 | not done |  | URT | None |
| 51E | 413 | B69 (94) | 55 (100) | URT | None |
| 51F | 468 | A29 (90) |  | URT | None |
|  |  |  |  |  |  |
| 52A |  | A76 (94) |  | LRT | None |
| 52B | 30 | not done |  | LRT | None |
| 52C | 50 | A76 (94) | 50 (100) | LRT | None |
|  |  |  |  |  |  |
| 53C |  | B93 (93) |  | URT | None |
| 53D | 14 | not done |  | URT | None |
| 53E | 29 | not done |  | URT | None |
| 53F | 56 | B93 (93) | 56 (100) | URT | Human adenovirus |
| 53G | 77 | B93 (93) | 77 (100) | URT | Human adenovirus, Coronavirus 229E |
|  |  |  |  |  |  |
| 55A |  | CoxA21 (98) |  | URT | None |
| 55B | 10 | not done |  | URT | None |
| 55C | 24 | CoxA21 (98) | 24 (100) | LRT | None |
| 55D | 55 | CoxA21 (98) | 55 (100) | URT | None |
| 55E | 93 | B14 (93) |  | LRT | None |
| 55F | 121 | B14 (93) | 28 (100) | LRT | None |

RSV: Respiratory syncytial virus

CoxA21 : Coxsackievirus A21

URT: Upper respiratory tract specimens are nasopharyngeal swabs or aspirates

LRT: Lower respiratory tract specimens are bronchoalveolar lavage fluids, bronchial or tracheal aspirates.

^a^ Patients are numbered and specimens labelled in with letters in alphabetical order. A corresponds to the first specimen, B to the second specimen, etc. In patient 53, the first two specimens were unavailable for typing, therefore only specimens 53C to G are reported.

^b^ the percentage of nucleic acid sequence identity compared to the nearest type is indicated

^c^ the percentage of nucleic acid sequence identity compared to the sequence of the prior specimen with the same type is indicated
