# Supplementary material for: In Vivo Persistence of Human Rhinoviruses in Immunosuppressed Patients
Source: PLoS One. 2017 Feb 2;12(2):e0170774. doi: 10.1371/journal.pone.0170774 (PMC5289482; doi:10.1371/journal.pone.0170774)
Supplement: S1 Text — (DOC) [file pone.0170774.s004.doc]

**S1 Text. GenBank accession numbers of the sequences obtained in this study**

KT920465, KT920466, KT920467, KT920468, KT920469, KT920470, KT920471, KT920472, KT920473, KT920474, KT920475, KT920476, KT920477, KT920478, KT920479, KT920480, KT920481, KT920482, KT920483, KT920484, KT920485, KT920486, KT920487, KT920488, KT920489, KT920490, KT920491, KT920492, KT920493, KT920494, KT920495, KT920496, KT920497, KT920498, KT920499, KT920500, KT920501, KT920502, KT920503, KT920504, KT920505, KT920506, KT920507, KT920508, KT920509, KT920510, KT920511, KT920512, KT920513, KT920514, KT920515, KT920516, KT920517, KT920518, KT920519, KT920520, KT920521, KT920522, KT920523, KT920524, KT920525, KT920526, KT920527, KT920528, KT920529, KT920530, KT920531, KT920532, KT920533, KT920534, KT920535, KT920536, KT920537, KT920538, KT920539, KT920540, KT920541, KT920542, KT920543, KT920544, KT920545, KT920546, KT920547, KT920548, KT920549, KT920550, KT920551, KT920552, KT920553, KT920554, KT920555, KT920556, KT920557, KT920558, KT920559, KT920560, KT920561, KT920562, KT920563, KT920564, KT920565, KT920566, KT920567, KT920568, KT920569, KT920570, KT920571, KT920572, KT920573, KT920574, KT920575, KT920576, KT920577, KT920578, KT920579, KT920580, KT920581, KT920582, KT920583, KT920584, KT920585, KT920586, KT920587, KT920588, KT920589, KT920590, KT920591, KT920592, KT920593, KT920594, KT920595, KT920596, KT920597, KT920598, KT920599, KT920600.
